# Ethical implications of *ex vivo* organ assessment and repair centers

**DOI:** 10.3389/frtra.2023.1184439

**Published:** 2023-07-11

**Authors:** Bryan A. Whitson, Sylvester M. Black

**Affiliations:** ^1^ Department of Surgery, The Ohio State University Wexner Medical Center, Columbus, OH, United States; ^2^ The Davis Heart and Lung Research Institute, The Ohio State University Wexner Medical, College of Medicine, Columbus, OH, United States

**Keywords:** *ex vivo*, transplantation, access, allocation, machine perfusion

## Introduction

Through *ex vivo* organ perfusion (EVOP), transplantation is poised for advancement not seen since the advent of cyclosporin (1). One may ask how? Why? What? The advances that EVOP will facilitate is through the development of organ assessment and repair centers (ARCs) (2, 3). These organ ARCs may be in an academic medical center, organ procurement organizations (OPO), or private industry. With this evolution, we need to ask what do EVOP and organ ARCs mean for the future of transplantation (Table 1)?

**Table 1:** Questions to ponder in expanded EVOP use.

• Access • Are candidate recipients only offered EVOP based on their geography and local center? • How do we consider multiple listing? • How do we navigate geographic boundaries of allocation and distribution? • Equity • Is EVOP available based on payor? • Is EVOP available based on center? • Is EVOP available based on OPO? • Does a candidate recipient's severity of illness influence EVOP offering? • Risk • Risk to the recipient • Risk to another recipient if an organ is not offered or match terminated • Risk to the program • Informed consent • What does this mean for the donor and their advocate? • What truly is informed consent for the recipient? • How do we manage therapeutic interventions during EVOP? • Allocation • Who allocates? • When is allocation done? • If a center undertakes the risk does that influence the outcome? • Cost (broadly considered) • Center • Patient • Payor • Personnel (technical expertise, on call, perfusionists, OR personnel) • Durable good • Deferred OR cases (donor hospital and recipient hospital) • Travel

**Figure 1:**
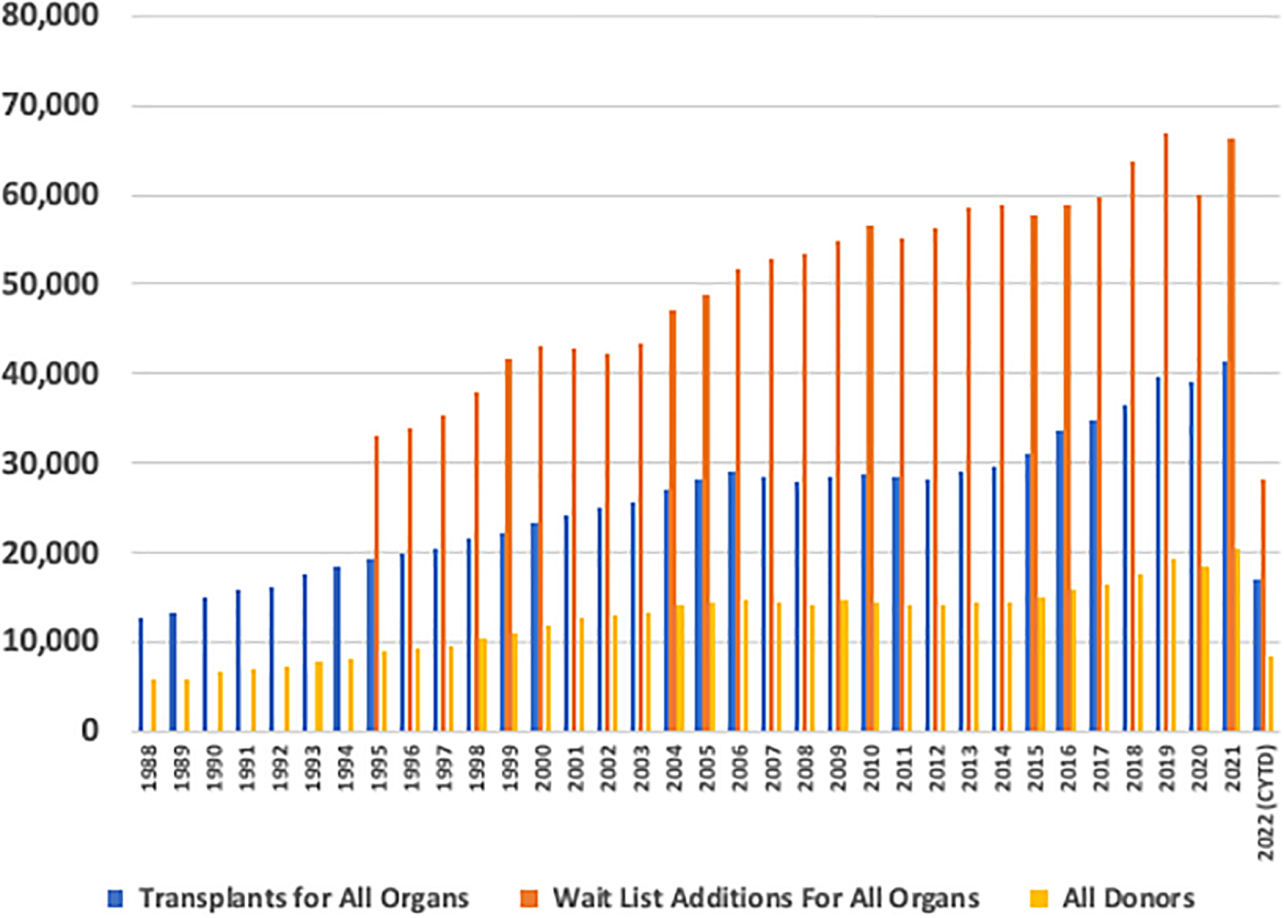
Annual United States transplant volume. Annual volume across the *y*-axis and transplant year across the *x*-axis. Total transplants performed, total waitlist additions, and total donors being recovered are denoted as annual bars.

The volume of transplants has essentially plateaued since approximately 2005 (4–10), predominantly from an inadequate number of available organs to meet the demand and benefits of transplantation (Figure 1). Transplant recipients demonstrate increased survival, compared to the waitlist for living and deceased donations. This is seen in every organ system, from the kidney to the intestine (10). As transplant practitioners know, we need to find a way to expand this life-saving and life-enhancing therapy to increase access to these donor organs, for potential patients to maximally benefit from transplantation.

## *Ex vivo* organ perfusion and transplant implications

Discussing the implications of EVOP on the future of transplantation, it is important to understand the history of organ support and how we arrived at its current state. We look back to circa 1935–1938 (11) with the Nobel Laureate Dr. Alexis Carrel and famed aviator Charles Lindberg. Charles Lindberg had a sister-in-law with heart failure (12), and he partnered with Alexis Carrel. They developed the “culture” of organs (13). Carrel was no stranger to the concept of cellular and tissue support outside of the body; indeed, his techniques were important in the development of the *in vitro* cell culture that is widely used in research today. In Carrel and Lindberg's estimation, the extrapolation of cell support could be applied to complex tissue, and even organs, and caught the public's attention, garnering the cover of *TIME* magazine with their devices, and theories were memorialized in their monograph, *The Culture of Organs*. Limited only by manufacturing and the equipment of their time (hand-blown glass), they maintained a cat's thyroid functioning for 18 days producing the thyroid hormone. While this is not the first time this concept has been conceptualized (14), Carrel and Lindberg's work is the first time it was able to be meaningfully reduced to practice with long-term application.

Today, EVOP re-emerged in clinical practice in 1999 by Stig Steen in Sweden, utilizing EVOP to maintain a lung outside the body—assessing, resuscitating, and transplanting the lungs to a recipient (15). Since then, EVOP has been utilized in every major organ transplant. EVOP in the liver, at least experimentally or in clinical trials, has resuscitated organs that previously were not considered transplantable. Steatotic livers have been perfused *ex vivo*, de-fatted, and resuscitated (16). In a similar fashion, lungs are cleansed of infectious disease (17), resuscitated across continents (18), and transplanted into highly sensitized recipients or crossmatch-positive recipients. We are able to 3D print organ scaffolds (19), decellularize human or non-human organs (19), and re-seed them followed by functional assessment before transplantation.

Hypothermic perfusion has been utilized for decades in kidney work and can potentially improve outcomes (20). This approach is widely used for cadaveric renal allografts before transplantation. There is a systemic review and meta-analysis of seven randomized controlled trials (RCTs) and 11 non-RCTs, looking at hypothermic machine preservation for kidneys. What we were able to find is that these organs stayed alive and that they had a trend towards decreased primary non-function. We also see this signal in normothermic *ex vivo* lung perfusion.

We have also entered an era of organ engineering—this is the repair or modification component. Within the next decade, we will have the ability to personalize an organ for a specific recipient. Whether that is decellularization and recellularization, gene or protein knock-in or knock-out, or building in conditional expression of immunosuppressant to minimize systemic toxicity, personalized experiences will increase organ availability and improve quality.

## Waitlist changes

When we look at lung transplantation, this organ system is the most mature in terms of numbers of transplants performed and overall experience with EVOP. Circa 2013, there were about 1,800 transplants and roughly 2,300 removals. For lung transplantation, death on the waiting list or removal is significant and even more profound in the pediatric world. Across all organs, about 1,900 pediatric candidates (aged <18 years) are listed for transplantation. Unfortunately, more pediatric patients were removed from the transplant list than transplanted, specifically for lungs. Of those patients on the list, 136 died, and others were removed. When we look at organ allocation for those patients who died on the list, potential lung recipients had the highest waitlist mortality at 38%.

Transplantation has variation (10, 21, 22); that is, variation in how and who is approached for donation, the process of dialogue, the variation in OPO timing and resuscitation, variation in transplant surgeon and transplant center risk tolerance, the variation in recipient illness, variation in geography, and we will have variation in access to EVOP and variation in thoughts on what organs to go to an organ ARC and who should or could receive the EVOP organ.

Expanding EVOP will need education, and involve partnering with donor hospitals, OPOs, and donor families to help describe the EVOP process, whether that is “research” or “standard of care”. Some patients are outside the traditional criteria for potential donation, whether they are elderly, have infectious diseases, have poor social situations, or have organs with pre-existing medical conditions. It is very hard to say, though it is clear that our historical thoughts on organ viability are changing. A large proportion of organs not being utilized is because no recipient could be found (the second largest at roughly 10%–15% of the total number of organ donors available) with approximately 70% are declined due to poor organ function (4–10). How we assess and recover those organs will rely on EVOP. Not every center or OPO has the technology across all organs, the bandwidth to conduct EVOP 24/7, or the programmatic tolerance to accept risk to the program and the receiving candidate. Our definition of a standard donor is changing, and we are moving toward more molecular diagnostics (23) and working to expand thoughts on what ischemic time means.

As we move further into the realm of machine perfusion, cost and access will be enormous concerns. The cost will be in terms of dollars and personnel. Some platforms will be portable, needing more extensive logistics, planes, and expertise that can travel. Some platforms will require additional personnel, such as perfusionists. For procurements being done locally where the platform does not travel, space and operating theater time are required. Not all centers will have the resources to deploy such teams or deploy them all of the time. Some perfusion platforms are quite expensive, particularly if the costs of the recovery surgeon are added on a la carte. These personnel, equipment, facilities, and time costs add up. Who will cover this bill? Will only those recipients with top-shelf coverage plans be allowed to see the benefit of EVOP? Will only those recipients listed at large centers be able to even have this as an option? How will a recipient know when these technologies are an option or not—not only when they are an option at the center, but when they are an option during the day or week?

Through these technologies, we can estimate that organ utilization rates could conceivably double. The United States XVIVO Perfusion HELP and NOVEL trials for lung donor resuscitation and expansion were able to recover more than half the lungs undergoing *ex vivo* lung perfusion (EVLP). With the United States Transmedics EXPAND trials in both the heart and lung, there is, in essence, no ischemic time. With the rethinking of cold ischemic time, and the pre-EVOP and post-EVOP cold ischemic times, or none at all, organs can go nearly anywhere in 6 h. When we can, essentially, double the number of organs available for transplantation and ischemic time is irrelevant, what does that allow us to do? It will enable us to go beyond the OPO level and look at national or international allocation beyond an arbitrary mileage radius. If donor organ shortage is not a concern, who *could* receive a transplant?

This concept was demonstrated by Drs. Love and Keshavjee with a lung transplant conducted internationally (18). In Chicago, a sick patient was at risk of imminent death. The donor lungs were marginal, demonstrating poor gas exchange. The recovered lungs were flown to Toronto, resuscitated for nearly 6 h, returned to Chicago, and implanted. The recipient did well with approximately 15.5 h of total ischemic time. When one puts that in comparison to a previous 4-h ischemic time mindset, it opens a broad geographic area for organ sharing. Time also allows the opportunity to find the best donor–recipient match to minimize rejection for those recipients fortunate enough or with the means to travel to be listed at a center with EVOP resources, technology, and expertise.

**Figure 2:**
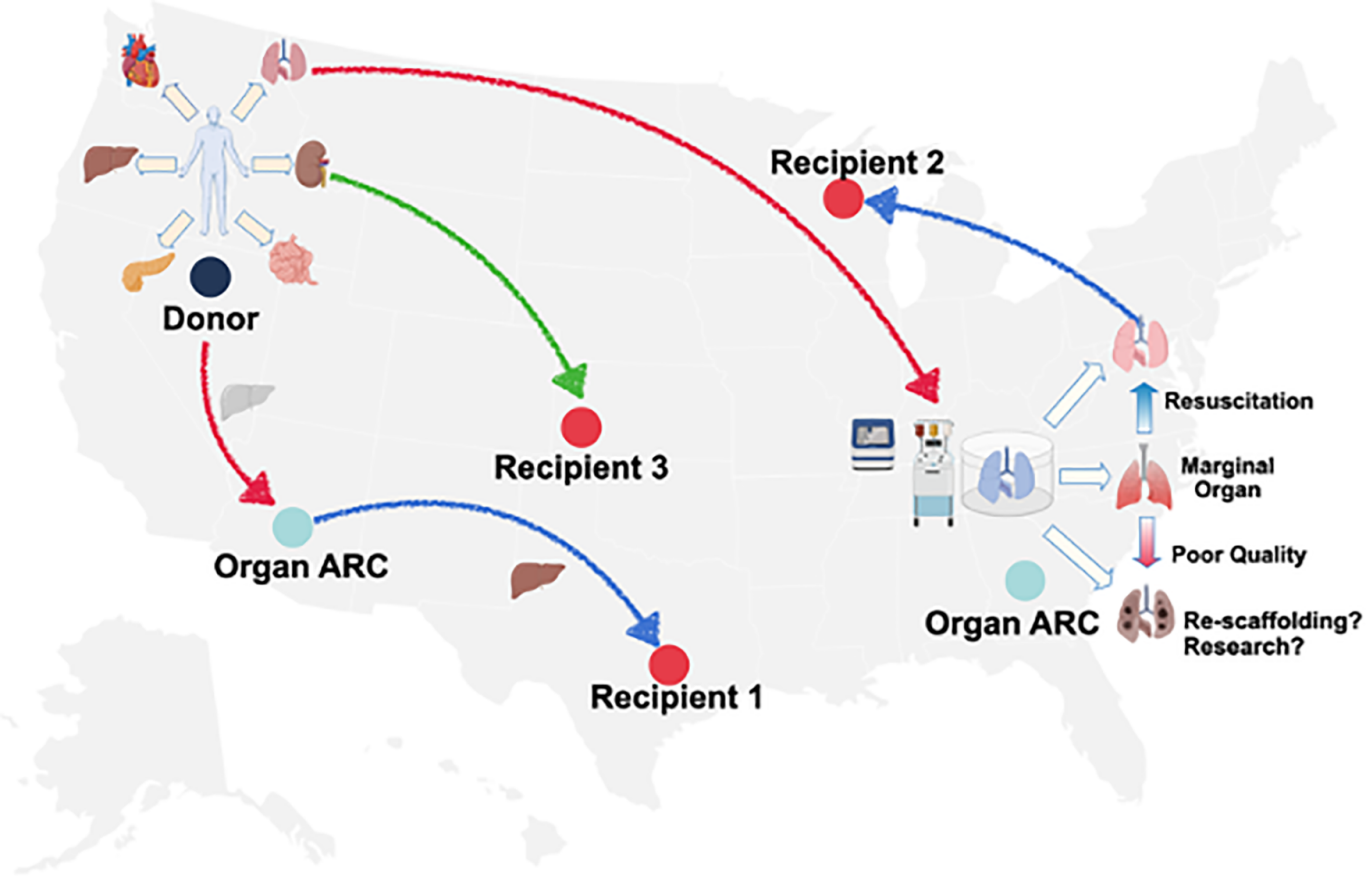
Schematic of organ assessment and repair centers and donor allograft utilization donor allografts recovered could, theoretically, be allocated outright, be transported via EVOP and undergo assessment, proceed to an organ ARC for EVOP, assessment and resuscitation and then proceed to transplantation or advance organ engineering research. Source: UNOS.org.

We are starting to see that the expertise and resources are clustered at some transplant centers, OPOs, or private entities. When they are clustered and done at scale, we begin to see organ ARCs. This also potentially takes our current allocation system and turns it on its head. Here are some questions: if one has a donor that may be in one region that goes to an organ ARC, the organ may get allocated to a recipient in that region (Figure 2). It also may be allocated to a different recipient in another region. It may go into the organ ARC, be resuscitated, and then reallocated to an entirely different recipient. No one specifically knows how the future will unfold. How and when do we allocate these organs? Is it once the match run is initially done or during or after the EVOP resuscitation? Is that EVOP match local, regional, or international?

In the medical community, we know that volume impacts outcomes for highly complex procedures. With that being the backdrop, who is best suited to do EVOP? Is that a small center, a large center, an OPO, or an industry? It is hard to say, although, conceptually, having concentrated expertise with a high number of consistent repetitions is likely the key to sustained success. This would argue for the regionalization of these technologies, which we have not embraced as a society in healthcare. Perhaps that is the role of a benevolent forward private industry model. These are concepts that we need to address.

Furthermore, who assumes the risk? We must be very mindful of all the things associated with EVOP done at scale. The risk (in all its facets), the cost (broadly speaking), and potential unintended consequences need to be considered. For some organs, primary non-function is catastrophic. There are no viable long-term bridges for a liver, heart, or lungs if the organ does not function. There are no good mechanical long-term bridges such as hemodialysis for the kidney. There are significant consequences for a non-functioning organ. Is that risk assumed by the center, the surgeon, or the OPO? Should there be accommodation for being innovative and advocating for transplantation in the current regulatory environment?

What is the cost, broadly speaking? The cost in terms of staffing may be high—we have teams going cross-country doing recoveries, as we saw in 2007 with an organ recovery team being lost in Lake Michigan. That is a considerable risk for the transplant team as they conduct recoveries. It costs money to fly to the recovery site, bring the organ back, and allocate it to the correct location. The perfusion packs and supplies associated with EVOP cost money. Staffing, operating theater time, all these things add up. Is this a matter of EVOP being available to people who have the ability to pay for their organs and then get the transplant, or is it a therapy that is a resource for all patients?

A bigger question is who pays if the organ does *not* get transplanted? What is an acceptable negative run rate for programs to remain viable? Will those serviced be underwritten by Centers for Medicare and Medicaid Services (CMS)? Does that fall to the recovering center? Does that return to OPOs on transplant waivers? If a center is looking at $40,000–$90,000 (for some organs) for resuscitation and EVOP, and if that organ is not transplanted, that can become a very costly endeavor. Over time, the cost likely will decrease, as has been seen with hypothermic kidney perfusion services.

Organ allocation is a question of access and equity (24). A few years ago, there was a lot of interest in pediatric patients being allowed access to the adult list. The outcomes of pediatric recipients are varied and the impact of waiting list time and allocation policies has a profound impact on these patient populations (25–31). It started a conversation about what does a “list” mean? Are we able to use the time that EVOP provides to truly run the list and conduct comprehensive tissue typing and allocate across geography? Does a list mean a national list? Does it send the best organ to the sickest recipient? Does one have to consider the initial allocation or the post-resuscitation score for the organ? If the initial accepting center is not able to get the organ back in sufficient quality, does it not get transplanted? Does an organ recovering approximately 75% of its function go to a less sick recipient? Does that calculus change on the organ and its ability to regenerate/recover *in vivo*? How we appropriate donor–recipient matching so everybody gets that access is the real challenge. When adult organs are transplanted into children, they have no adverse or deleterious effects and no change in their chronic graft rejections. Allocating adult organs to pediatric recipients may be a way to mitigate the dying on the waiting list (32). How does one assess priority? How does one assess equity?

Some EVOP allocation processes are nebulous with sizeable national variation, as demonstrated by the recent National Academies of Sciences, Engineering, and Medicine Report (24). Reassignment and match timing are legitimate concerns; for example, reassigning organs when a center accepts an organ for one recipient, calls in a back-up, and then decides that the organ is not able to be transplanted into the initial recipient and, due to timing and the inability to be reallocated, the organ goes into the back-up recipient because otherwise the organ will go to waste. Sometimes this is legitimate and OPOs grant back-up waivers. The center should and does know their list and what the recipient is able to tolerate—this is an art, the art of transplantation. If a center accepts an organ, and resuscitates it to the appropriate quality, are they able to manage and take that risk for their recipients' benefit? Do those organs go back and get reallocated, or are those organs for the center to allocate however they feel is most appropriate?

If a center resuscitates an organ to the point where they can manage the risk for a given recipient, is that appropriate? What if the neighboring center/surgeon/recipient cannot? Perhaps the center has the ability to reassign the organ to a less sick recipient who may be able to tolerate graft dysfunction?

How does regulatory oversight play into this? “Small” centers may not be able to manage the programmatic risk that comes with performing these transplants. A “big” center may have the ability to absorb a lot of risks and have the workforce to conduct the EVOP. There is also the risk tolerance of recipients. When we present these concepts to our recipients in the clinic and then at the time of transplant, they seldom say no. The receiving candidate may not completely understand the nuances of the process and informed consent may be nebulous. The dying or struggling patient often trusts in the physicians to use their best judgment in utilizing the organ as they want to be able to breathe, run, urinate, or not be jaundiced, depending on the organ and the disease process.

As we start to modify organs and engineer them to be more resistant to ischemia-reperfusion injury, or not be antigenic or not need as much immunosuppression, and then transplant them, we need to work to understand the known risks and theoretical risks. How do we advance the science of organs that have genetic modification (33) or have therapies delivered (16, 34, 35) to them when we are not able to conduct large-sized prospective RCTs? We do not know what effect these treatments will have, so how does one counsel the patients? Informed consent with EVOP and the organ ARC area is nebulous. What does “research” mean? How do we evaluate novel therapeutics?

Numerous biomolecules/drugs/approaches appear promising in small animal models, but how does that translate into people? How are trials performed when everything is off label? There is no way to do a prospective RCT in the EVOP sphere. Do we know who is getting EVOP and who is not? And then is transplantation becoming commercialized? Maybe. Timing does matter, and if we look at the current standard, we can keep organs viable for hours, although can we stretch those hours into days or a week (36), allowing us to change the organ for the better. There are those champions in academia and industry who believe in this technology and are driven to expand the field of transplantation to improve access and quality.

The impact is unclear as we move forward. However, when time counts, and we are trying to save lives, if industry is better suited, perhaps we should embrace it so that we can bring this technology and these organs to patients. At the end of the day, we all achieve more transplants with better outcomes. Whether this is done in academia in the OPOs or industry, does it matter if we are able to do more transplants and do them better? These are all concepts that we need to discuss.

Regardless of the “where” EVOP is performed, regardless of “what” platform is utilized for EVOP, and “what” organ ARC concept is utilized, this impactful technology will facilitate increased organ utilization. The much bigger “how” is how we, as a community, ensure transparency, advocacy, access, and equity for our donors and recipients.
